# Geldanamycin-mediated inhibition of heat shock protein 90 partially activates dendritic cells, but interferes with their full maturation, accompanied by impaired upregulation of RelB

**DOI:** 10.1186/1756-9966-33-16

**Published:** 2014-02-13

**Authors:** Stefanie Trojandt, Angelika B Reske-Kunz, Matthias Bros

**Affiliations:** 1 Department of Dermatology, Medical Center of the Johannes Gutenberg-University, Mainz, Germany

**Keywords:** Heat shock protein 90, Geldanamycin, NF-κB, RelB, Dendritic cell, T cell

## Abstract

**Background:**

The chaperon heat shock protein 90 (HSP90) constitutes an important target for anti-tumor therapy due to its essential role in the stabilization of oncogenes. However, HSP90 is ubiquitously active to orchestrate protein turnover, chemotherapeutics that target HSP90 may affect immune cells as a significant side effect. Therefore, we asked for potential effects of pharmacological HSP90 inhibition at a therapeutically relevant concentration on human dendritic cells (DCs) as main inducers of both cellular and humoral immune responses, and on human CD4^+^ T cells as directly activated by DCs and essential to confer B cell help.

**Methods:**

Unstimulated human monocyte-derived DCs (MO-DCs) were treated with the prototypical HSP90 inhibitor geldanamycin (GA). Based on dose titration studies performed to assess cytotoxic effects, GA was applied at a rather low concentration, comparable to serum levels of clinically used HSP90 inhibitors. The immuno-phenotype (surface markers, cytokines), migratory capacity, allo T cell stimulatory and polarizing properties (proliferation, cytokine pattern) of GA-treated MO-DCs were assessed. Moreover, effects of GA on resting and differentially stimulated CD4^+^ T cells in terms of cytotoxicity and proliferation were analysed.

**Results:**

GA induced partial activation of unstimulated MO-DCs. In contrast, when coapplied in the course of MO-DC stimulation, GA prevented the acquisition of a fully mature DC phenotype. Consequently, this MO-DC population exerted lower allo CD4^+^ T cell stimulation and cytokine production. Furthermore, GA exerted no cytotoxic effect on resting T cells, but abrogated proliferation of T cells stimulated by MO-DCs at either state of activation or by stimulatory antibodies.

**Conclusion:**

HSP90 inhibitors at clinically relevant concentrations may modulate adaptive immune responses both on the level of DC activation and T cell proliferation. Surprisingly, unstimulated DCs may be partially activated by that agent. However, due to the potent detrimental effects of HSP90 inhibitors on stimulated CD4^+^ T cells, as an outcome a patients T cell responses might be impaired. Therefore, HSP90 inhibitors most probably are not suitable for treatment in combination with immunotherapeutic approaches aimed to induce DC/T cell activation.

## Background

The ubiquitously expressed chaperon heat shock protein 90 (HSP90), often acting together with other chaperons like HSP70, binds to a variety of newly synthesized proteins to support their proper folding and to prevent aggregation and interaction with third party proteins [1]. By now, several hundreds of HSP90 client proteins have been identified, including a number of protooncogenes [2]. Based on the vital role of HSP90 to stabilize mutated oncogenic proteins and to promote accumulation of over-expressed oncogenes [3], and its high level expression in tumor cells [4], this chaperone has gained long-standing interest as a molecular target for cancer therapy [5]. In this regard, the prototypic HSP90 inhibitor geldanamycin (GA) exerted strong proapoptotic effects on tumor cells *in vitro*[6]. Derivatives of GA [7], and other HSP90 inhibitors [8], which are optimized in terms of metabolic stability and reduced hepato-toxicity, are being tested in several clinical trials [9].

In light of the essential role of HSP90 in protein homeostasis in all cell types [10], it is of vital importance to elucidate consequences of drug-mediated inhibition of HSP90 on the patients’ immune system as required to eradicate drug-resistant tumor cells [11]. In this respect, dendritic cells (DCs) as the main inducers of primary immune responses play an essential role [12]. Stimulation of DCs by pathogen-derived molecular patterns and endogenous danger signals as well as by activated T cells results in the activation and upregulated expression of NF-κB transcription factors like RelB [13], which in turn orchestrate expression of genes required for functional DC maturation [14]. Inhibition of HSP90 by GA was shown to result in diminished NF-κB activity in tumor cells due to impaired functional activity of NF-κB signaling molecules [15-17]. This suggests a modulatory role of HSP90 for the DC activation state.

Here we show that treatment of MO-DCs with GA at low concentration (0.1 μm) resulted in their partial activation. In contrast, GA interfered with stimulation of MO-DCs. In addition, GA prevented the proliferation of stimulated T cells. These findings suggest that inhibition of HSP90 may differentially affect the DC activation state as well as T cell responses in individuals treated with HSP90-inhibiting chemotherapeutics.

## Methods

### Cell culture

Peripheral blood mononuclear cells (PBMCs) were derived from buffy coats of healthy donors by Ficoll density gradient centrifugation, and monocytes were isolated by plastic adherence for 1 h in 6-well tissue culture plates (Starlab, Hamburg, Germany) as described [18]. Monocytes were differentiated in culture medium (Gibco, Houston, TX), containing 2% (v/v) heat-inactivated (56°C, 30 min) autologous plasma, penicillin (100 U/ml)/streptomycin (100 μg/ml) (both PAA, Pasching, Austria), supplemented with recombinant human (rh) GM-CSF (200 U/ml, Berlex, Seattle, WA), IL-4 (1,000 U/ml; ImmunoTools, Friesoythe, Germany). Cells were fed with fresh medium every other day. On 6 of culture, part of the differentiated MO-DCs was treated with GA (Alexis Biochemicals, Lausen, Switzerland) at the concentrations indicated, and aliquots were stimulated with a cocktail of proinflammatory mediators (each 10 ng/ml of rh IL-1β and rh TNF-α (PeproTech, Hamburg, Germany, and 1 μg/ml prostaglandin E2 (PGE_2_, Alexis Biochemicals) for two days [18,19]. Cell lines HEK293T [20] and IGROV1 [21] were cultured as described.

### Cytotoxicity assays

Cells (MO-DCs: 2×10^5^, HEK293T and IGROV1: 5×10^4^, CD4^+^ T cells, prepared as outlined below: 5 × 10^5^) were seeded into wells of 96-well cell culture plates (Starlab) in a volume of 100 μl of their respective culture medium, and GA was added at various concentrations as indicated. Aliquots of MO-DCs were supplemented with stimulation cocktail in addition. Two days later, an MTT assay was performed as recommended by the supplier (Promega, Madison, WI).

### Proliferation assays

CD4^+^ T cells were enriched from PBMCs by positive immunomagnetic separation (MACS, Miltenyi Biotec). CD4^+^ T cells (10^5^) were cocultured with titrated numbers of allogenic MO-DCs in 96-well plates (Greiner Bio-One, Frickenhausen, Germany) in triplicates in 200 μl of culture medium for 5 days. In some experiments, CD4^+^ T cells were stimulated with anti-CD3 (1 μg/ml) plus anti-CD28 (0.5 μg/ml) antibodies (both from BioLegend, San Diego, CA) for 5 days, in the absence or presence of GA (0.1 μM). T cell proliferation was assessed by genomic incorporation of [^3^H] thymidine (0.25 μCi/well) added for the last 16 h of culture, measured in a liquid scintillation counter (1205 Betaplate, LKB Wallac, Turcu, Finnland).

### Cytokine detection

Supernatants of DC cultures were harvested on day 8, and of DC/T cell cocultures on day 5, and contents of IL-5, IL-6, IL-12p40, and INF-γ were measured by ELISA as recommended (all ELISA Kits from eBioscience, San Diego, CA).

### Flow cytometry

Harvested cells (5×10^5^) were incubated for 20 min at 4°C with antibodies: fluorescein isothiocyanate (FITC)-conjugated anti-HLA-DR (L243), phycoerythrin (PE)-cyanine 5-conjugated anti-CD80 (2D10), allophycocyanin-conjugated anti-CD86 (IT2.2) (all from BioLegend), PE-conjugated anti-CD83 (HB15e; BD Pharmingen, San Diego, CA), and corresponding isotype controls, respectively. Afterwards, washed DCs were analysed in a FACSCalibur (BD Biosciences, Franklin Lakes, NJ) equipped with CELLQUEST software (BD). For intracellular detection of Fascin 1 (Fscn1), MO-DCs were permeabilized with methanol (10 min on ice), washed with pre-cooled PBS, and incubated with FITC-conjugated anti-Fscn1 (55 K-2; Dako, Glostrup, Denmark) or isotype control antibody. All samples were analysed at the same fluorescence detector settings in order to allow for direct comparison of mean fluorescence intensities (MFIs).

### Migration assays

To prepare 100 μl of DC-loaded collagen matrices, first 5 μl of 7.5% (w/v) Na_2_CO_3_ and 10 μl of 10× MEM (Invitrogen) were mixed, and then added to 75 μl of PureCol*^®^* bovine collagen I (Invitrogen). Afterwards, 67 μl of this mixture was further mixed with 33 μl of cell suspension containing 3 × 10^5^ DCs, loaded onto a glass slide covered with a cover slip, and incubated at 37°C for 45 min to allow for gelation. IMDM supplemented with penicillin/streptomycin was then added on top of the collagen gel. Spontaneous migration of MO-DC populations was monitored for about 6 h in 2 min intervals by time-lapse microscopy with a BX61 microscope (UAPO lens 20×/340, NA 0.75), equipped with a FView camera (all Olympus, Hamburg, Germany) using Cell^P^ software (SIS, Münster, Germany).

### Promoter reporter assays

HEK293T cells were seeded in wells of a 6 well cluster plate (Greiner), and were transfected at a confluence of about 90%. Cells were transfected in parallel with transcription factor (TF) responsive luciferase reporter vectors (pAP1-luc, pCRE-luc, pISRE-luc, pNFAT-luc, pNF-κB-luc, and promoterless negative control; all from Agilent, Palo Alto, CA). For transfection, plasmid DNA (4 μg) was complexed with Fugene HD (2 μl; Promega) for 20 min as recommended by the manufacturer. 5 hr after transfection, cells were harvested and were equally split into wells of a 24 well cluster plate (Greiner). On the following day, triplicates were treated with GA and/or the MO-DC maturation cocktail. One day later, cells were harvested, lysed in passive lysis buffer (Promega), and assayed for luciferase detection in a Turner Designs TD-20/20 luminometer (Promega). Luciferase activities were normalized by the activity of the promoterless reporter.

### Western blot analysis

MO-DCs (≥ 1 × 10^6^) were lysed with RIPA buffer (1% (v/v) NP-40, 1% (v/v) sodium deoxycholate, 0.1% (w/v) SDS, 0.15 M NaCl, 0.01 M Na_3_PO_4_, 2 mM EDTA, 1 mM dichlorodiphenyltrichloroethane, 0.2 mM Na_3_VO_4_, 50 mM NaF, 100 U/ml aprotinin, 1 mM phenylmethylsulfonyl fluoride, and 1% (v/v) of Complete Protease inhibitor cocktail (Roche Diagnostics, Mannheim, Germany). Protein concentrations were quantified by Bradford protein assay (Bio-Rad, Munich, Germany), and 30 μg of protein per sample were assayed. Protein samples were separated on a 10% (w/v) sodium dodecyl sulphate-polyacrylamide gel, and transferred to a nitrocellulose membrane (GE Healthcare Europe, Freiburg, Germany). Western blots were probed with rabbit polyclonal antibodies specific for human p65 NF-κB (C22B4), phospho-p65 NF-κB (Ser536; 93H1), both from Cell Signaling Technology (Boston, MA), RelB (C-19; Santa Cruz Biotechnology, CA), ß-actin (Abcam, Cambridge, UK), and with mouse anti human monoclonal antibody specific for IκB-α (L35A5), followed by incubation with a secondary goat antibody (anti-rabbit or anti-mouse IgG), conjugated with horseradish peroxidase (all from Cell Signaling Technology). ECL plus staining (PerkinElmer, Waltham, MA) served as substrate for horseradish peroxidase.

### Statistics

Data are given as mean ± SEM. Statistically significant differences were analysed by applying the Student’s two-tailed *t* test.

## Results

### GA promotes expression of activation markers by unstimulated MO-DCs, but interferes with their stimulation-induced upregulation

**Figure 1:**
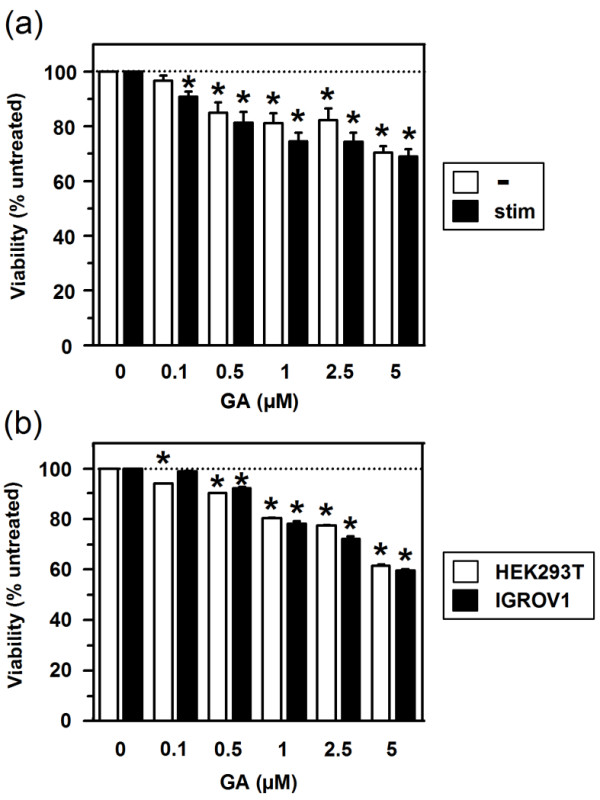
**GA affects the viability of MO-DCs at either state of activation as well as cancer cells to a similar extent. (a)** MO-DCs on day 6 of culture, and **(b)** HEK293 and IGROV1 cells were treated with GA at the concentrations indicated for 48 h in triplicates. One h after application of GA, aliquots of MO-DCs were stimulated with the stimulation cocktail (see Methods) in addition. **(a, b)** Cell viability was quantified by MTT assay. Viability of untreated cells was arbitrarily set to 100%. Data represent means ± SEM of two (HEK293), three (IGROV1), and four (MO-DCs) independent experiments. Statistical significance: *versus untreated cells. For reasons of clarity, the degree of statistical significance is not further delineated (*P < 0.05).

Due to the pronounced proapoptotic effect of the HSP90 inhibitor GA, we first assessed cytotoxicity of this agent on MO-DCs. As shown in Figure 1a, treatment of MO-DCs with GA for 48 h resulted in impaired viability in a dose-dependent manner to a similar extent when applied to MO-DCs at either unstimulated state or when coadministered with the stimulation cocktail. Sensitivity of MO-DCs to the cytotoxic effect of GA was comparable to that of the the immortalized cell line HEK293T, derived from embryonic kidney cells, and IGROV1, an ovarian adenocarcinoma line (Figure 1b). A concentration of 0.1 μM GA, which only slightly affected viability of both MO-DC populations, was used in further experiments.

**Figure 2:**
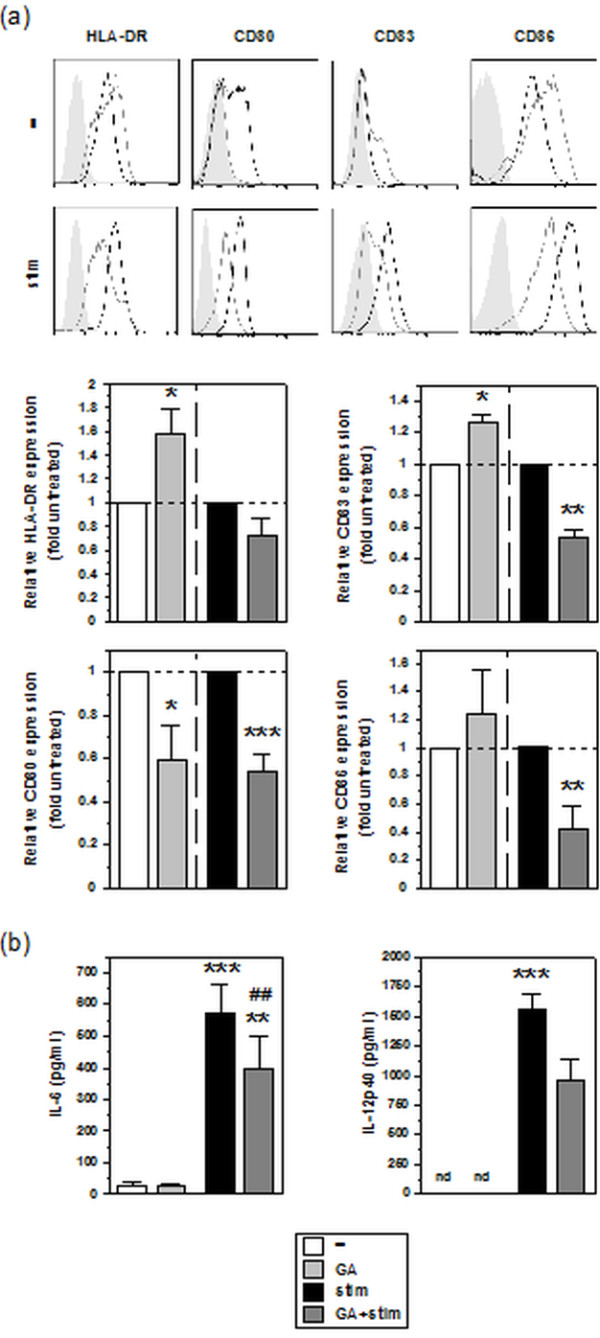
**GA affects the phenotype of MO-DCs in a gene-dependent manner.** Aliquots of MO-DCs on day 6 of culture were differentially treated with GA (0.1 μM) and/or stimulation cocktail (see Methods section) as indicated for 48 h. **(a)** The expression levels of the markers indicated were assessed by flow cytometry. Upper panel: Marker expression was detected in unstimulated (-) and cocktail-stimulated (stim) MO-DCs left untreated (dark line) or treated with 0.1 μM GA (light grey). Shaded area: isotype control of MO-DCs left untreated (corresponding isotype controls of GA-treated MO-DCs were comparable). Graphs are representative of 4-5 independent experiments each. Lower panel: Relative expression intensities of DC surface marker expression are given as mean fluorescence intensities (MFIs), normalized to the MFI of unstimulated or stimulated MO-DCs left untreated. Data represent the means ± SEM of 4-5 independent experiments each. **(b)** Contents of IL-6 and IL-12p40 in the supernatants of harvested MO-DC populations were assayed by ELISA. Data represent means ± SEM of 10 independent experiments each. nd: not detectable. Statistical significance: **(a)** *versus untreated MO-DCs, **(b)** *versus unstimulated untreated MO-DCs, ^#^GA-treated at stimulated versus unstimulated state. **(a, b)** *P < 0.05, ^##,**^P < 0.01, ^***^P < 0.001.

Next, we asked for effects of GA on the immuno-phenotype of MO-DCs. At unstimulated state, treatment of MO-DCs with 0.1 μM GA resulted in moderately upregulated expression of HLA-DR, CD83, and CD86, albeit not significant in case of the latter. CD80 surface expression on the other hand was attenuated (Figure 2a; Additional file 1: Table S1). In response to treatment with a stimulation cocktail (IL-1ß, TNF-α, and PGE_2_), MO-DCs upregulated expression of either monitored marker to a significant extent, except for CD80 (Additional file 1: Table S1). However, cotreatment of MO-DCs with GA during stimulation resulted in profound inhibition of all activation-associated DC surface markers monitored.

MO-DCs at an unstimulated state expressed the proinflammatory cytokines IL-6 and IL-12 at low levels, but at high extent after stimulation (Figure 2b). GA treatment alone exerted no effect on the production of either mediator by MO-DCs under basal conditions. However, when coapplied during stimulation, GA attenuated the otherwise activation-associated increase of either cytokine. Taken together, these findings suggest that GA differentially affects the immuno-phenotype of MO-DCs, depending on their state of activation.

### GA impairs the migratory capacity of MO-DCs

**Figure 3:**
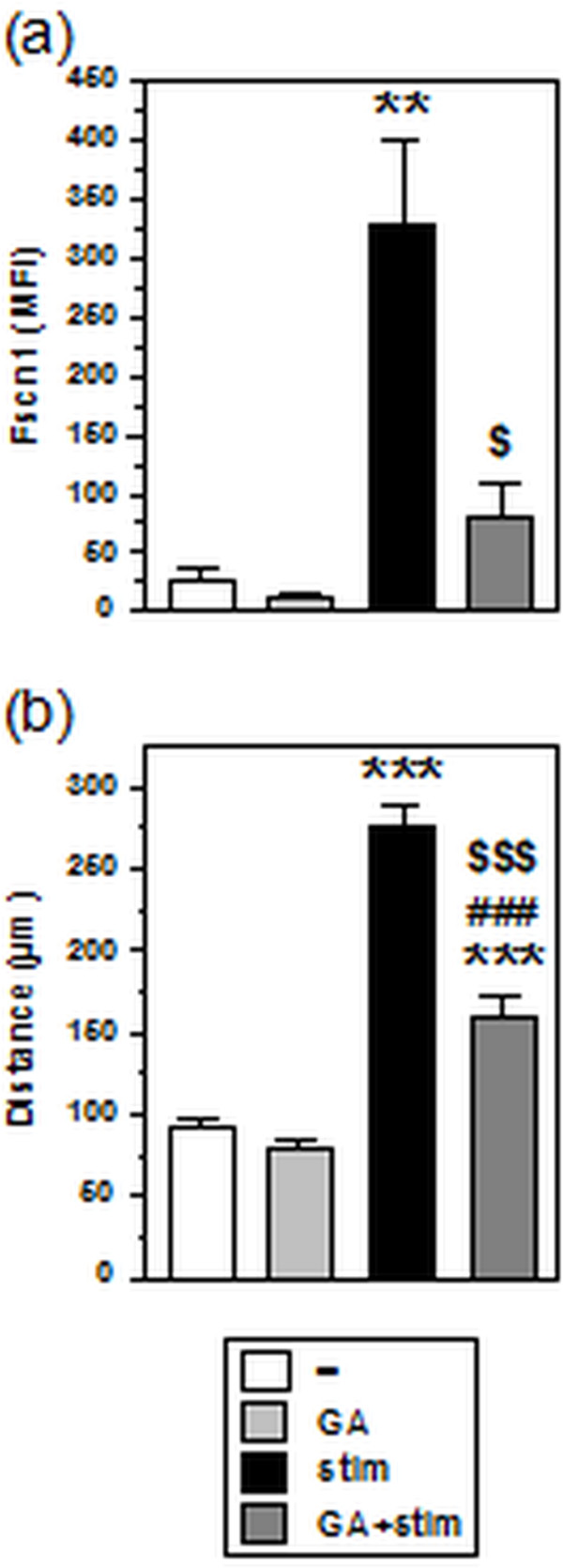
**GA impairs the migratory activity of stimulated MO-DCs.** Groups of MO-DCs were generated as described (see legend of Figure 2). **(a)** Expression of the actin-bundling protein Fscn1 was assessed by intracellular flow cytometry. Data represent the means ± SEM of MFI intensities of 6 independent experiments. **(b)** Spontaneous migration of MO-DC populations in 3D collagen matrices was monitored for 6 h by time lapse analysis in intervals of 2 min. Graphs represent the means ± SEM of around 80 MO-DCs per group individually tracked in two independent experiments compiled. Statistical significance: *versus unstimulated MO-DCs, ^#^GA-treated MO-DCs at stimulated versus unstimulated state, and ^$^GA-treated versus untreated MO-DCs at stimulated state (^*,$^*P* < 0.05, **P < 0.01, ^***,###,$$$^*P* < 0.001).

Enhanced migratory activity constitutes another hallmark of activated DCs. This functional property is regulated in part by the actin-bundling protein fascin (Fscn)1 [22], which also serves to promote DC/T cell interaction as a prerequisite for T cell stimulation [23]. Expression of Fscn1 in unstimulated MO-DCs was slightly reduced after treatment with GA, and its stimulation-associated upregulation was strongly inhibited in MO-DCs cotreated with GA during stimulation (Figure 3a). These results suggested detrimental effects of GA on the cytoskeletal plasticity of MO-DCs, which in turn may alter their migratory capacity. To this end, we performed migration assays in 3D collagen gels, intended to mimic the in vivo environment [24]. Unstimulated MO-DCs were not affected by GA pretreatment in their spontaneous migration in terms of distance covered during the time monitored (Figure 3b). While stimulated MO-DCs were characterized by an enhanced mobility, cotreatment with GA during stimulation resulted in a diminished migratory activity in terms of distance covered and speed.

The endocytotic capacity, which is characteristic of unstimulated DCs, is downregulated upon activation. Unstimulated MO-DCs pretreated with GA showed lower endocytotic uptake of FITC-labeled dextran than untreated MO-DCs, albeit not significant (Additional file 2: Figure S1). This finding is in line with the notion that GA affects the activation state of unstimulated MO-DCs to a moderate extent.

### GA diminishes the T cell activation capacity of stimulated MO-DCs

**Figure 4:**
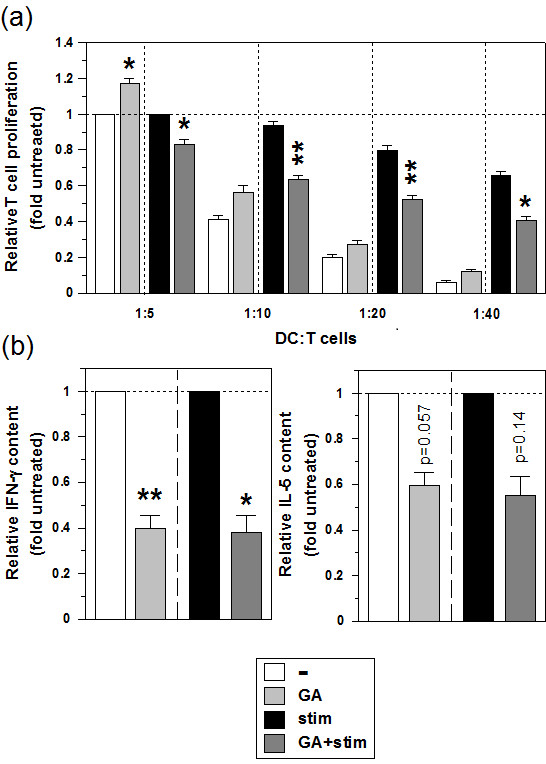
**GA impairs the T cell activation capacity of stimulated MO-DC.** Groups of MO-DCs were generated as described (see legend of Figure 2). **(a)** Titrated numbers of the various MO-DC populations (starting at 2 × 10^4^ cells, two-fold diluted) were cocultured with allogenic CD4^+^ T cells (10^5^) in triplicates for 4 days. T cell proliferation was assessed by uptake of [^3^H] thymidine during the last 16 h of culture. CD4^+^ T cell proliferation as induced by unstimulated or stimulated MO-DCs left untreated employed at the highest DC number was arbitrarily set to one in each experiment. Graphs show the means ± SEM of 3 independent experiments compiled. **(b)** Supernatants of day 4 DC/T cell cocultures (ratio 1:5) were assayed for cytokine contents by ELISA. Graphs show relative cytokine levels, normalized to the levels of unstimulated or stimulated MO-DCs left untreated. Data represent the means ± SEM of 7 independent experiments each. Statistical significance: **(a)** *GA-treated versus untreated MO-DCs; **(b)** *versus unstimulated untreated MO-DCs (*P < 0.05, **P < 0.01).

Due to the differential effects of GA on the immuno-phenotype of unstimulated and stimulated MO-DCs, we assessed their T cell stimulatory capacity. For this, differentially treated MO-DC populations were cocultured with allogenic CD4^+^ T cells, and both T cell proliferation and the cytokine pattern in DC/T cell cocultures were analyzed. Unstimulated MO-DCs exerted a moderate allogenic T cell stimulatory capacity, while stimulated MO-DCs mediated strong T cell proliferation (Additional file 3: Figure S2). Unstimulated MO-DCs pretreated with GA, in line with partially enhanced expression of activation markers, elicited slightly higher allogenic T cell proliferation than untreated MO-DCs. In contrast, MO-DCs pretreated with the stimulation cocktail plus GA exhibited a significantly impaired allogenic T cell stimulatory capacity as compared with the corresponding control (Figure 4a). This finding corresponds with the attenuated expression of activation markers due to interference of GA with DC stimulation.

Cocultures that containd untreated MO-DCs were characterized by low contents of the Th1 marker IFN-γ and of the Th2 cytokine IL-5, and both cytokines were present at strongly enhanced levels in DC/T cell cocultures which contained stimulated MO-DCs (Additional file 3: Figure S2b). Pretreatment of unstimulated and stimulated MO-DCs with GA resulted in reduced production of IFN-γ and IL-5 in DC/T cell cocultures as compared with the corresponding controls (Figure 4b).

Taken together, so far these results show that GA interferes with the stimulation-induced activation of MO-DCs in terms of immuno-phenotype, migration, and T cell stimulatory capacity. In contrast, unstimulated MO-DCs are partially activated in response to treatment with GA.

### GA affects distinct signalling pathways, and inhibits stimulation-induced upregulation of RelB in stimulated MO-DCs

Next we analysed the outcome of GA-mediated inhibition of HSP90 on the level of transcription factor (TF) activities as the downstream effectors of cellular signalling. Due to the ubiquitous activity of HSP90, and since MO-DCs are rather refractory towards non-viral transfection and may be partially activated in response to transfection [25], we used HEK293T cells for these analysis. HEK293T cells were transfected with several TF-responsive luciferase reporter vectors, and rested prior to treatment with GA and/or the MO-DC stimulation cocktail, whose components have been shown to stimulate this cell line (IL-1ß, and TNF-α [26]; PGE_2_[27]).

**Figure 5:**
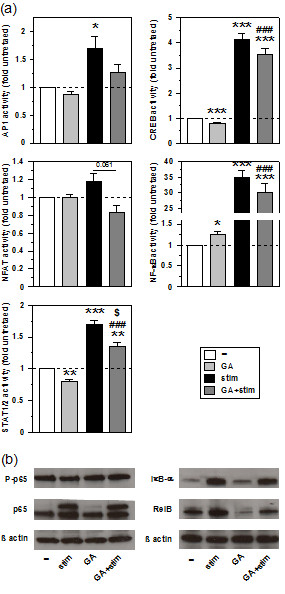
**GA affects TF activities, and reduces RelB expression in MO-DCs. (a)** HEK293T cells were transfected with TF responsive luciferase reporter vectors. After 5 h, cells were split, and aliquots were differentially treated in triplicates with GA, and/or the MO-DC maturation cocktail as indicated. One day later, luciferase activities were detected. Data show the means ± SEM of three experiments, normalized to the relative luciferase activity of untreated HEK293T cells, arbitrarily set to 1. Statistical significance: *versus unstimulated untreated, and ^#^GA-treated at stimulated versus unstimulated state, and ^$^GA-treated versus untreated at stimulated state (*^,$^*P* < 0.05, **P < 0.01, ***^,###^*P* < 0.001). **(b)** Groups of MO-DCs were generated as described (see legend of Figure 2). Derived protein (each 30 μg) was separated on SDS-PAGE, and western blots were performed. β-actin served as loading control. The graph is representative of two independent experiments.

Under basal conditions, GA treatment exerted either no (AP1, NFAT) or slightly inhibitory (CREB, STAT1/2) effects on the TFs monitored (Figure 5a). Only activity of NF-κB was moderately enhanced by GA. Stimulation with the maturation cocktail had no effect on NFAT activity, but resulted in moderate upregulation of AP1, STAT1/2, and CREB activity, as well as in pronounced augmentation of NF-κB activity. Cotreatment with GA during stimulation had no major effect on the enhanced activity of CREB and NF-κB, but impaired AP1, and STAT1/2 activities.

These findings indicate that HSP90 affects the activities of distinct TFs at basal conditions, and in response to stimulation. In light of the well acknowledged importance of NF-κB activity for the DC activation process, and the finding that GA evoked slightly elevated NF-κB activation under basal conditions, we asked for effects of GA on NF-κB regulation in MO-DCs. The ubiquitous NF-κB family member p65 is upregulated in stimulated DCs [13,28], and its transient activation is reflected by phosphorylation of Ser536 [29]. GA treatment exerted no major effect on the expression level of p65 and the fraction of phosphorylated protein in unstimulated MO-DCs (Figure 5b, left panel). Stimulation of MO-DCs resulted in an increase of p65, as reflected by the arisal of a second band, to a similar extent in both untreated and GA-treated cells. The fraction of Ser536-phosphorylated p65 was unaltered, most probably due to the rather long period of stimulation. We also monitored expression of the ubiquitously expressed endogenous NF-κB inhibitor IκB-α, which is degraded immediately after stimulation of DCs, but strongly upregulated at later time points to limit NF-κB activation [30]. In line, MO-DCs stimulated for 48 h, displayed higher IκB-α levels than unstimulated MO-DCs (Figure 5b, right panel). GA treatment mediated no alterations of IκB-α levels in MO-DCs at either state of activation. While both p65 and IκB-α are expressed in a ubiquitous manner, the NF-κB family member RelB is confined to professional antigen presenting cells (APCs), upregulated in response to stimulation [28]. RelB has proven essential for the acquisition of a mature DC activation state [31], which prompted us to monitor its expression. As expected, unstimulated MO-DCs expressed RelB at low level, which was increased following stimulation (Figure 5b, right panel). GA treatment of unstimulated MO-DCs yielded a reduced RelB content as compared with untreated MO-DCs. When applied in the course of stimulation, GA prevented the otherwise stimulation-associated increase in RelB expression.

These findings indicate that GA may affect the activities of a number of TFs. These TFs are known to contribute to determine the state of activity of DCs. In this context, NF-κB may play an important role as highlighted by impaired RelB expression in MO-DCs treated with GA in the course of stimulation.

### GA does not exert cytotoxic effects on resting T cells, but abrogates their stimulation-induced proliferation

**Figure 6:**
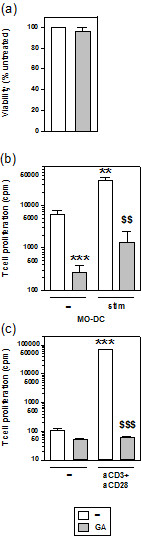
**GA impairs the proliferation of stimulated CD4**^**+ **^**T cells.** CD4^+^ T cells were assayed for effects of GA on their **(a)** viability, and **(b, c)** stimulation-induced proliferation. **(a)** CD4^+^ T cells (5×10^5^) were supplemented with rhIL-2 (20 U/ml), seeded in triplicates, and aliquots were treated with 0.1 μM GA. After 48 h, viability was assessed by MTT assay. Viability of untreated cells was arbitrarily set to 100%. Data represent means ± SEM of two independent experiments. **(b, c)** CD4^+^ T cells (10^5^) were stimulated **(b)** by allogenic MO-DCs (2×10^4^) at unstimulated (-) or stimulated state (stim), and **(c)** by anti-CD3 (1 μg/ml) plus anti-CD28 antibodies (0.5 μg/ml). T cell proliferation was determined by incorporation of [^3^H] thymidine for the last 16 h of culture. Data represent the means ± SEM of three independent experiments each. Statistical significance: **(b)** *versus unstimulated MO-DCs, ^$^versus stimulated MO-DCs without GA, **(c)** *versus unstimulated T cells, ^$^versus stimulated T cells without GA (**^,$$^*P* < 0.01, ***^,$$$^*P* < 0.01).

Finally, we investigated whether GA besides its detrimental effects on MO-Cs may also directly modulate T cell activation. Resting T cells were not affected in their viability upon treatment with GA (Figure 6a). Activated allogenic MO-DCs induced higher levels of T cell proliferation than unstimulated MO-DCs (Figure 6b). When GA was added to these cocultures, the proliferative potential of T cells stimulated by either MO-DC population strongly dropped. In this setting, GA may affect T cell activation/proliferation directly, but also indirectly by inhibiting MO-DC functions. Therefore, T cells were also stimulated in a DC-independent manner by applying T cell-activating antibodies. Polyclonal stimulation resulted in a strong T cell proliferative response, which was completely abrogated in the presence of GA (Figure 6c).

These results indicate that GA may hamper the induction of adaptive immune responses both on the level of DC activation as well as T cell stimulation and/or proliferation.

## Discussion

Here we show that the prototypic HSP90 inhibitor GA exerted cytotoxic effects on human MO-DCs both at unstimulated state as well during stimulation in a dose-dependent manner. We chose a concentration of GA (0.1 μM) devoid of detrimental effects on the viability of MO-DCs to analyze the influence of this agent on the immuno-phenotype and functions of MO-DCs. Of note, this concentration broadly corresponds to plasma levels of GA-derived HSP90 inhibitors used in the course of treatment of patients in clinical trials [32,33].

Unstimulated MO-DCs treated with GA were characterized by differential regulation of DC surface markers: While CD80 expression levels were reduced, HLA-DR, CD83, and CD86 were upregulated. In accordance with the elevated expression of the latter markers, whose expression is controlled in part by NF-κB [14], we noted moderately enhanced NF-κB activity in GA-treated HEK293T cells, which may explain in part the enhanced state of activation of likewise treated MO-DCs. However, neither the expression level of the endogenous NF-κB inhibitor IκB-α [34], nor the level and activation state of the ubiquitously expressed NF-κB family member p65 [35] were altered in GA-treated MO-DCs. Moreover, expression of the largely APC-restricted NF-κB family member RelB [36] was actually reduced in this MO-DC population. Therefore, further analysis is required to elucidate whether GA treatment results in activation of NF-κB in unstimulated MO-DCs, and which of the other members of this TF family [13] may be involved. Besides, the partial activation of GA-treated MO-DCs may also result at least in part from reduced activity of signaling pathways and TFs that serve to maintain a low state of activation in DCs. For example, agents that activate the cAMP/PKA signaling axis also induce a largely maturation-resistant DC activation state [37]. In this regard, we observed moderate down-regulation of CREB activity in GA-treated HEK293T cells, and it remains to be analyzed whether impaired CREB activity in turn may favour DC activation.

In striking contrast to our findings of enhanced expression of some DC activation markers in GA-treated MO-DCs, Bae and coworkers [38] observed profound down-regulation of HLA molecules as well as of all costimulators monitored in MO-DCs subjected to treatment with GA. This discrepancy may be due to GA dose effects, since Bae and coworkers used a tenfold higher dose of GA (1 μM) [38] than employed by us, which in their study was the only dose tested on MO-DCs to assess apoptosis-inducing effects.

Unstimulated DCs are specialized in the uptake of antigen by various means, including receptor-mediated endocytosis, but cease to engulf antigen upon stimulation [39]. Both in our study and in the report of Bae and coworkers [38] GA-treated MO-DCs were characterized by slightly decreased endocytotic activity. The finding of a GA-dependent decrease in antigen uptake by MO-DCs supports the notion of a partially activated state. Alternatively, it is possible that proteins involved in receptor-mediated endocytosis may constitute genuine HSP90 client proteins, affected by GA-mediated HSP90 inhibition. Interestingly, HSP90 is required for transfer of internalized antigen from the endosome to the cytosol for subsequent cross-presentation [40].

In our study, unstimulated GA-treated DCs displayed a slightly enhanced allo CD4^+^ T cell stimulatory capacity. This finding may be explained in part by the moderately upregulated expression of HLA-DR and of CD83 as well as by the tendency of elevated CD86 surface levels in GA-treated MO-DCs. This may may compensate for the impaired expression of CD80, in order to facilitate elevated antigen presentation and T cell stimulation.

In marked contrast to the partially stimulatory effects of GA on unstimulated MO-DCs, this agent interfered with the stimulation-associated increase in surface expression of all DC activation markers monitored, as well as proinflammatory mediators, while HLA-DR remained largely unaffected. In case of CD80, the only molecule diminished in expression by GA treatment in unstimulated MO-DCs, GA completely abrogated the otherwise stimulation-associated increase in surface expression. This finding suggests that CD80 may be regulated in a qualitatively distinct manner as compared with the other markers assessed. Similarly, Bae and coworkers reported on lower expression of all DC markers monitored for MO-DCs treated with GA (1 μM) during stimulation [38]. However, in that study, effects of GA on DC surface marker expression were not assessed side-by-side in unstimulated and stimulated MO-DCs, given that expression levels of most activation markers shown were lower in stimulated than in unstimulated control MO-DCs. Therefore, it remains unclear whether treatment of MO-DCs with GA at that high dose abolished stimulation-dependent upregulation of surface markers, or only partially inhibited upregulation, as was observed for most molecules in our work for a ten-fold lower dose of GA applied.

In agreement with impaired upregulation of the cytoskeletal protein Fscn1, required for dendrite formation [22] and migration [41], MO-DCs cotreated with GA in the course of stimulation were characterized by a lower migratory activity than the corresponding control group. This functional defect may reflect in part impaired actin polymerization, shown to require HSP90 activity [42].

MO-DCs treated with GA during stimulation, in accordance with reduced upregulation of DC activation markers and proinflammatory cytokines, exhibited lower allo CD4^+^ T cell activation capacity as compared with stimulated control MO-DCs. Consequently, the corresponding DC/T cell cocultures contained lower levels of the Th1/Th2 effector cytokines [43] IFN-γ, and IL-5.

In general, stimulation of MO-DCs results in the activation of a number of signaling pathways, and a number of key regulators have been reported to constitute client proteins of HSP90. In this regard, STAT1 has been identified as a genuine HSP90 target [44]. Here we show that GA-treated HEK293T cells displayed impaired STAT1/2 activity under basal conditions, and impaired upregulation in response to stimulation. In stimulated DCs, STAT1 has been demonstrated to mediate increased expression of activation markers like CD40 [45], and its inhibition may contribute to impaired DC maturation.

Moreover, MAPK members JNK [46], and p38 [47] have been shown to positively regulate DC activation, and both kinases interact with HSP90 (JNK [48], p38 [49]). Both MAPK are known to activate PKC, which in turn mediates phosphorylation-dependent activation of TFs of the AP-1 family that are important i.e. for expression of MMP-9 in stimulated DCs as a prerequisite for emigration from the periphery [50]. In line with the relevance of HSP90-mediated protein maturation of either MAPK, we observed impaired upregulation of AP-1 activity in HEK293T cells cotreated with GA and the maturation cocktail. Besides, stimulation-dependent MAPK activation is known increase of NF-κB activity [13], based on transient degradation of the endogenous inhibitor IκB-α [34], and in case of APCs also on elevated expression and activity of the NF-κB family member RelB [51]. In case of DCs, RelB is essential for stimulation-dependent increases of activation marker expression and consequently the T cell stimulatory capacity [33]. Therefore, our finding of GA-dependently impaired RelB expression in stimulated Mo-DCs may explain in part the detrimental effects of this agent on the phenotype and function of stimulated Mo-DCs. In HEK293T cells, GA-treatment mediated no detrimental effect on the stimulation-associated increase in NF-κB activity, which may be explained by the APC-specific character of RelB expression [51]. However, in previous studies inhibition of HSP90 by GA was shown to diminish NF-κB activity in tumor cells due to impaired expression of the NK-κB signaling regulators IKK [15], NIK [16], and RIP1 [17]. Limited activity of either regulator may contribute to attenuated RelB expression in stimulated MO-DCs cotreated with GA.

In T cells GA may inhibit the expression of the tyrosine kinase lck, and impair its stimulation-induced phosphorylation as evidenced in a human T cell line (Jurkat) [52,53]. Due to this early block in T cell activation, IL-2 production of stimulated T cells was largely abrogated. Most recently, GA was demonstrated to affect as well the expression of several T cell receptor-associated molecules, namely TCRαß, CD4 and CD28 [54]. In accordance, GA prevented the proliferation of lymphocytes treated with stimulatory antibodies [53] and of T cells stimulated by either MO-DCs or mitogen [54]. In line, we observed largely abrogated proliferation of CD4^+^ T cells stimulated by unstimulated or stimulated MO-DCs or by application of stimulatory antibodies.

## Conclusions

Our study has shown that GA-mediated inhibition of HSP90 in unstimulated MO-DCs may result in partial activation of the cells by yet unknown mechanisms. On the other hand, GA treatment impaired MO-DC stimulation and largely abrogated both polyclonal and DC-mediated T cell proliferation. Chemotherapeutics that act to inhibit HSP90 may therefore exert rather inhibitory effects on the patients’ immune system, and most likely are not preferable for combination with immunotherapy that targets the DC/T cell axis to mount potent anti-tumor responses.

## Competing interests

The authors declare that they have no competing interests.

## Authors’ contributions

ST and MB performed the experiments. MB and ABRK designed the study. ST, MB, and ABRK wrote the paper. All authors read and approved the final manuscript.

## Supplementary Material

Additional file 1: Table S1 GA affects surface marker expression by MO-DCs in an activation state-dependent manner. Click here for file

Additional file 2: Figure S1 GA slightly reduces the endocytotic activity of unstimulated MO-DCs. Click here for file

Additional file 3: Figure S2 MO-DCs acquire potent T cell stimulatory capacity in response to stimulation. Click here for file
