# Supplementary material for: Geldanamycin-mediated inhibition of heat shock protein 90 partially activates dendritic cells, but interferes with their full maturation, accompanied by impaired upregulation of RelB
Source: J Exp Clin Cancer Res. 2014 Feb 13;33(1):16. doi: 10.1186/1756-9966-33-16 (PMC3926270; doi:10.1186/1756-9966-33-16)
Supplement: Additional file 1: Table S1 — GA affects surface marker expression by MO-DCs in an activation state-dependent manner. [file 1756-9966-33-16-S1.doc]

**Additional file**

**Table 1.** **GA affects surface marker expression by MO-DCs in an activation state-dependent manner**

| **Surface marker** | **MO-DC population** | | | |
| --- | --- | --- | --- | --- |
|  | **-** | **GA** | **stim** | **GA+stim** |
| HLA-DR | 38.98±6.98 | 58.78±8.53 | 89.5±13.11 ***** | 59.83±5.73 |
| CD80 | 10.72±2.29 | 6.23±1.74 | 23.7±8.28 | 14.23±6.83 |
| CD83 | 8.47±0.83 | 10.74±1.19 | 39.4±5.18 ***** | 20.38±3.64 |
| CD86 | 102.45±35.01 | 125.29±36.53 | 642.5±193.27 ***** | 239.37±114.12 |

MFI values of surface marker expression by the different MO-DC populations shown in a normalized manner in Fig. 2a are given as mean ± SEM of 4-5 experiments each. Asterisks denote statistically significant (*P* < 0.05) differences of untreated MO-DCs at stimulated versus unstimulated state.
