# Supplementary figures and images for: Geldanamycin-mediated inhibition of heat shock protein 90 partially activates dendritic cells, but interferes with their full maturation, accompanied by impaired upregulation of RelB
Source: J Exp Clin Cancer Res. 2014 Feb 13;33(1):16. doi: 10.1186/1756-9966-33-16 (PMC3926270; doi:10.1186/1756-9966-33-16)

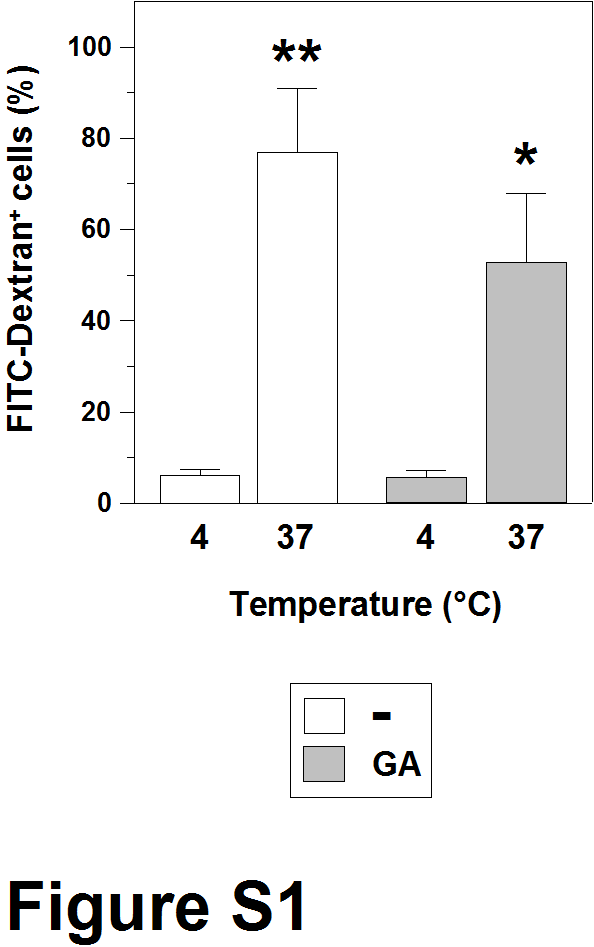

Supplement: Additional file 2: Figure S1 — GA slightly reduces the endocytotic activity of unstimulated MO-DCs. [file 1756-9966-33-16-S2.tiff]

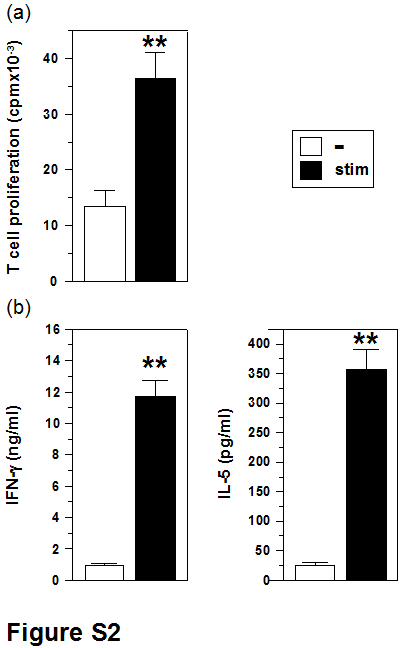

Supplement: Additional file 3: Figure S2 — MO-DCs acquire potent T cell stimulatory capacity in response to stimulation. [file 1756-9966-33-16-S3.tiff]
